# Nodular Elastosis of the Pancreas

**DOI:** 10.1155/2015/521959

**Published:** 2015-08-09

**Authors:** Whitney Wedel, Geoffrey Talmon, Aaron Sasson

**Affiliations:** ^1^Department of Pathology and Microbiology, University of Nebraska Medical Center, Omaha, NE 68198, USA; ^2^Department of Surgery, University of Nebraska Medical Center, Omaha, NE 68198, USA

## Abstract

Elastofibromatous change is a benign process that has been increasingly recognized in the tubular gastrointestinal tract. These changes can present as a colonic polyp or be seen in conjunction with inflammatory changes. Similar lesions have been noted in the liver, apparently associated with vascular injury. We describe a case in which multiple circumscribed nodules of elastofibromatous change within the pancreas had a similar morphology to nodular elastosis of the liver. To our knowledge, this is the first description of such a finding occurring within the pancreas.

## 1. Introduction

Mesenchymal lesions of the pancreas are uncommon. While the gamut of spindle cell tumors seen at other sites may involve the organ, collectively they account for 1-2% of pancreatic neoplasms [1]. While being well recognized in the skin and soft tissue, elastofibromatous change (EFC) is an under recognized, benign finding in specimens from the gastrointestinal system that characteristically display a localized focal or diffuse increase in elastin fibers and collagen, often in association with blood vessels [2]. The lesion has been favored to represent a reactive alteration by some authors.

Areas demonstrating EFC have been described in the mucosa of the colon, small bowel, and stomach, where the lesion has earned the moniker “elastofibroma.” EFC has also been seen within the parenchyma of the liver and only rarely within the pancreas. We present a peculiar elastin-rich lesion that occurred in the neck of the pancreas of a middle-aged woman that, to our knowledge, has yet to be described and we dub “nodular elastosis.”

## 2. Case Report

The patient is a 44-year-old female with a history of a possible inflammatory arthritis who presented with complaints of abdominal fullness and early satiety. There was no family history or clinical stigmata of a connective tissue disease. She had slightly elevated liver transaminase levels (alanine aminotransferase: 89 U/L (ref: 14–54 U/L)) and underwent ultrasonography to evaluate possible gallbladder disease which disclosed a mass in the neck of the pancreas. A follow-up CT scan demonstrated the presence of two well-circumscribed nodules (approximately 1.4 cm and 0.6 cm). No evidence of adenopathy or liver lesions was identified.

Upon further evaluation, the patient reported symptoms of periodic diaphoresis and tremulousness. The patient, a nurse by profession, had measured her blood glucose levels during these episodes and found them to be as low as 60 mg/dL; her symptoms resolved with oral glucose intake. She underwent an endoscopic ultrasound which confirmed the presence of at least one hypoechoic pancreatic mass, and an associated fine needle aspiration was nondiagnostic.

The patient was admitted to the hospital as an in-patient and underwent a 72-hour glucose fasting test, and multiple serum glucose levels were determined to be 40 mg/dL (reference range: 70–125 mg/dL) which prompted consideration of a pancreatic endocrine tumor. However, insulin levels were appropriately suppressed [9 mcIU/mL (reference range: <18 mcIU/mL)], and C-peptide levels were low as well [0.2 ng/mL (reference range: 1.0–7.6 ng/mL)], a finding felt to be inconsistent with an insulinoma. Due to the presence of the pancreatic masses, exploratory laparotomy and surgical resection of the neck of the pancreas with a central pancreatectomy and a Roux-en-Y pancreaticojejunostomy to the distal pancreatic remnant were performed.

**Figure 1:**
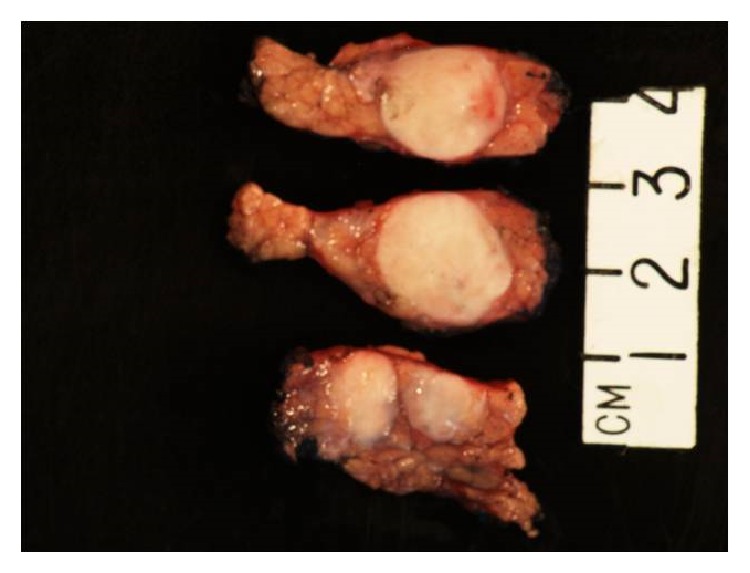
Gross photograph of circumscribed, firm nodules that are well demarcated from the surrounding unremarkable pancreatic parenchyma.

**Figure 2:**
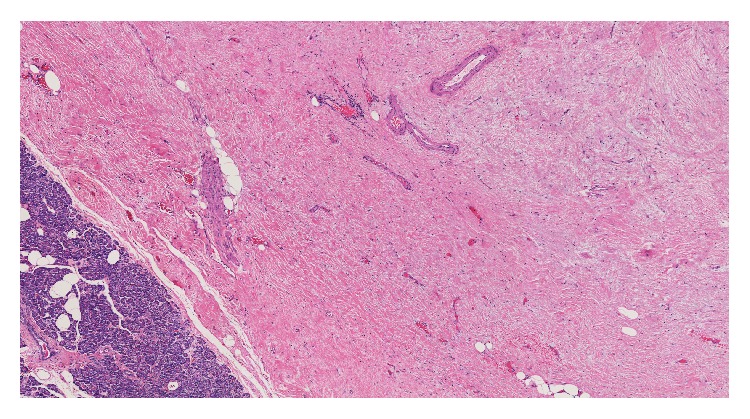
The hypocellular nodules demonstrated abundant collagen with nondescript bland spindle cells and occasional small indwelling arteries (H&E, 40x).

**Figure 3:**
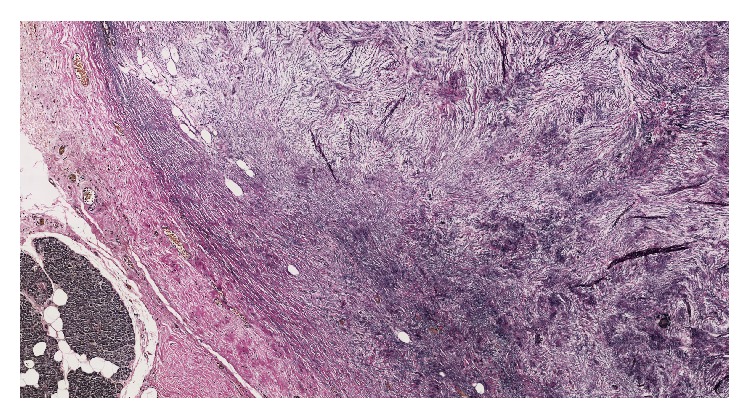
Each lesion was richly invested by bundles of elastic fibers (VVG, 40x).

Gross examination revealed three discrete, well-circumscribed, tan-white nodules (1.6 cm, 1.0 cm, and 0.3 cm in diameter, Figure 1) completely confined to the pancreatic parenchyma. The surrounding pancreatic parenchyma was completely unremarkable. Histologically, the nodules were comprised of hypocellular collagen with abundant VVG-positive elastic fibers (Figures 2 and 3), admixed with rare bland spindle to stellate cells without indwelling large vessels. Immunoperoxidase staining for insulin, glucagon, and somatostatin showed benign entrapped islets within the nodules. At the periphery of the nodules were additional rare foci of entrapped benign-appearing acini and islets, acinar ductal metaplasia, and occasional hemosiderin-laden macrophages. Storiform fibrosis and venular phlebitis were not observed, nor was there mitotic activity, cellular atypia, or necrosis. The spindle cells were negative for epithelial membrane antigen (EMA), H-caldesmon, pancytokeratin, CD117, S100, and desmin. No nuclear staining with beta catenin was identified. Staining for IgG and IgG4 did not disclose an increase in plasma cells with either marker. The patient is free of recurrence of metastasis after 12 months. She has continued monitoring her blood glucose at home and has not reported any values less than 60 mg/dL, similar to those values seen before pancreatic resection.

## 3. Discussion

EFC has been documented at multiple sites within the gastrointestinal tract. Proximally located lesions often have a nondescript endoscopic appearance and are likely underrecognized. Within the oral cavity, EFC may present as leukoplakia and have an inconsistent association with prior trauma [3]. EFC involving the stomach and small bowel are often seen by endoscopists as nonspecific areas of ulceration, hemorrhage, or mucosal/mural thickening [4, 5]. Overall, within the tubular gastrointestinal tract, an association with benign conditions (previous biopsy or irradiation, gastric ulcer, and atrophic gastritis) has been documented [6].

In contrast, EFC in the colon more often presents as a discrete polypoid lesion and, as such, is the best characterized EFC. It predominantly involves the submucosa and occasionally the muscularis mucosa with sparing of the mucosa in the majority of cases. Some have theorized that such lesions may result from prior clinically apparent or subclinical inflammatory injury, possibly related to degeneration of blood vessels [7]. It has been shown that injured adult tissue can produce disorganized elastic fibers in excessive amounts which may be synthesized by a variety of mesenchymal cells [8]. Foci of EFC demonstrate an increase in fine fibrillar elastic fibers with a distinct association with submucosal vessels, some of which were obliterated. By H&E staining, the grey appearance of the elastic tissue and the association with blood vessels may be mistaken for amyloidosis. In a review by Märkl et al., the authors advocate using the term “gastrointestinal angioelastosis,” as their contention is that EFC represents a form of disordered healing after vascular injury [2].

A spectrum of EFC has been recently found to occur in the liver and shares many of the histologic features as in the tubular gastrointestinal tract. These changes have been associated with segmental atrophy and are often subcapsular and associated with abnormally thick-walled and/or thrombosed blood vessels, which may indicate vascular injury as an inciting event. Some lesions demonstrate an appearance dubbed “nodular elastosis” [9]. In these cases, the zone(s) of elastosis were sharply demarcated from the parenchyma with an abrupt interface with the adjacent hepatocytes. Indwelling vessels were not described.

The findings in our case most closely resemble nodular elastosis of the liver. Although no associated thrombosed and/or thick-walled vessels were identified, this association has been inconsistent in EFC throughout the GI tract and has not been described as a feature in hepatic nodular elastosis. Hobbs and colleagues posit that progressive degeneration and elastin deposition may obscure central vessels. It is also possible that, as with the hepatic nodular elastosis and EFC at other sites, the etiology is related to prior inflammatory damage or segmental parenchymal atrophy supported by the presence of multiple hemosiderin-laden macrophages near the periphery of the pancreatic parenchyma adjacent to the lesions.

We present a unique and to our knowledge heretofore undescribed, nodular elastotic lesion within the pancreas that bears a resemblance to nodular elastosis of the liver. This report expands the gastrointestinal sites in which EFC may be seen and pathologists should consider the lesion in their differential diagnoses of nonepithelial pancreatic tumors.

In this case, the definitive cause of the patient's mild hypoglycemia remains unexplained. As previously stated, the patient underwent an initial 72-hour glucose fasting test, and multiple serum glucose levels were determined to be 40 mg/dL (reference range: 70–125 mg/dL), with insulin and C-peptide levels appropriately albeit incompletely suppressed, with an insulin: glucose ratio of 0.18, findings felt to be inconsistent with an insulinoma. Additionally, during the 72-hour glucose fasting test, the patient did not experience substantial symptoms of hypoglycemia, suggesting that the relative hypoglycemia during the fast may have been physiologic, with incomplete suppression of insulin and C-peptide. Another possible consideration for the cause of mild hypoglycemia was adrenal insufficiency, secondary to the patient's frequent but inconsistent use of corticosteroids to treat her arthritis. Overall, the patient's symptomatology and home blood glucose levels in the months following surgery have remained similar to those seen before pancreatic resection. While it is also possible that the pancreatic lesions represent prominent regression of a pancreatic endocrine tumor which was not identified despite thorough sampling, the lack of change in the patient's postoperative glucose levels argues against this.
